# PRIMA-1 inhibits Y220C p53 amyloid aggregation and synergizes with cisplatin in hepatocellular carcinoma

**DOI:** 10.3389/fmolb.2023.1165132

**Published:** 2023-04-10

**Authors:** Mariana M. Paz, Giulia D. S. Ferretti, Mafalda M. C. Martins-Dinis, Beatriz I. S. Ferreira, Amanda Faier-Pereira, Thibaut Barnoud, Otacilio C. Moreira, Jerson L. Silva, Yraima Cordeiro, Luciana P. Rangel

**Affiliations:** ^1^ Faculty of Pharmacy, Federal University of Rio de Janeiro, Rio de Janeiro, Brazil; ^2^ Institute of Medical Biochemistry Leopoldo de Meis, National Institute of Science and Technology for Structural Biology and Bioimaging, National Center of Nuclear Magnetic Resonance Jiri Jonas, Federal University of Rio de Janeiro, Rio de Janeiro, Brazil; ^3^ Department of Biochemistry and Molecular Biology, Medical University of South Carolina, Charleston, SC, United States; ^4^ Real Time PCR Platform RPT09A, Laboratory of Molecular Virology and Parasitology, Oswaldo Cruz Institute, Oswaldo Cruz Foundation, Rio de Janeiro, Brazil

**Keywords:** p53, amyloid aggregation, PRIMA-1, hepatocellular carcinoma, cisplatin

## Abstract

Hepatocellular carcinoma (HCC) is the third leading cause of cancer-related deaths worldwide. Although many therapeutic options are available, several factors, including the presence of p53 mutations, impact tumor development and therapeutic resistance. *TP53* is the second most frequently mutated gene in HCC, comprising more than 30% of cases. Mutations in p53 result in the formation of amyloid aggregates that promote tumor progression. The use of PRIMA-1, a small molecule capable of restoring p53, is a therapeutic strategy to pharmacologically target the amyloid state mutant p53. In this study, we characterize an HCC mutant p53 model for the study of p53 amyloid aggregation in HCC cell lines, from in silico analysis of p53 mutants to a 3D-cell culture model and demonstrate the unprecedented inhibition of Y220C mutant p53 aggregation by PRIMA-1. In addition, our data show beneficial effects of PRIMA-1 in several “gain of function” properties of mutant-p53 cancer cells, including migration, adhesion, proliferation, and drug resistance. We also demonstrate that the combination of PRIMA-1 and cisplatin is a promising approach for HCC therapy. Taken together, our data support the premise that targeting the amyloid-state of mutant p53 may be an attractive therapeutic approach for HCC, and highlight PRIMA-1 as a new candidate for combination therapy with cisplatin.

## Introduction

Hepatocellular carcinoma (HCC) is the most frequently occurring primary cancer that affects the liver, comprising approximately 90% of cases (Llovet et al., 2021). It is the third leading cause of cancer-related deaths worldwide (Sung et al., 2021) and, without proper treatment, leads to a median survival of only 6 months (Mezale et al., 2018). Therapeutic options for HCC include liver-directed therapy, surgical resection, liver transplantation, and systemic therapy (Kim et al., 2017). However, despite numerous therapeutic options, tumor detection in an advanced stage, the existence of other concomitant diseases, and the presence of mutations in tumor cells, such as p53 mutations, favor tumor development and therapeutic resistance (Chen et al., 2003; Kunst et al., 2016; Caron de Fromentel and Levrero, 2020).

p53 is a tumor suppressor protein, also known as the “guardian of the genome,” since it is related, directly or not, to several functions to prevent DNA damages that promote cancer tumorigenesis (Vogelstein et al., 2000; Barnoud et al., 2021; D’Orazi, 2021). Unfortunately, *TP53* remains the most frequently mutated gene in human cancer, affecting more than 50% of cancer cases (Rivlin et al., 2011; Chillemi et al., 2013). In HCC, it is the second most common mutation, comprising more than 30% of cases (Lee, 2015). Missense mutations are the most frequent type (approximately 80%) (Kastenhuber and Lowe, 2017; Boettcher et al., 2019). The six most prevalent missense mutations in HCC occur in hotspots within the DNA-binding domain (DBD) of p53, which are codons 249, 273, 248, 175, 245, and 282 (Caron de Fromentel and Levrero, 2020).

Mutations in p53 generally promote a dominant-negative (DN) effect: when a mutation in one allele of the *TP53* gene occurs, it leads to the formation of a tetramer containing both versions of the protein, mutant (mutp53) and wild-type (WTp53), thus preventing its interaction with DNA and blocking its tumor suppressive role in transcriptional regulation, which, in turn, severely hinders the cell’s ability to control its growth and proliferation, leading to tumorigenesis (Morgunkova et al., 2003; Stein et al., 2020; Gerasimavicius et al., 2022). In some instances, mutp53 can also acquire new oncogenic properties known as “gain-of-function” (GoF) properties, which add functionalities to increase tumor malignancy. GoFs in mutp53 promote a more aggressive phenotype within the tumor, including enhanced proliferation and invasion; in addition, they deregulate metabolic pathways and promote metastasis and drug resistance (Silva et al., 2014; Zhang et al., 2016).

One explanation for the DN effect of p53 involves the ability of mutp53 to form aggregates. In this scenario, the mutant *TP53* allele leads to the synthesis of a less stable protein that has an increased propensity to form amyloids. This phenomenon can lead to the sequestration of WTp53, in turn promoting its inactivation (Schilling et al., 2010; Ano Bom et al., 2012; Silva et al., 2013; Stein et al., 2020; Li et al., 2022). Several p53 mutants cause protein aggregation with different conformational states, from oligomers to amyloid fibrils. Similar to prions found in neurodegenerative diseases, a so-called prion-like effect occurs by leading the amyloid aggregates of mutp53 to convert WTp53 into an aggregated form, which may suspend its tumor suppressor functions and accumulate in the cell (Ishimaru et al., 2003; Ano Bom et al., 2012; Silva et al., 2014; Navalkar et al., 2020). Additionally, studies show that these aggregates can be transmitted to other cells, probably contributing to cancer progression (Forget et al., 2013; Rangel et al., 2014; Silva et al., 2014; Burdakov et al., 2018; Iwahashi et al., 2020; Navalkar et al., 2021).

The small molecule “p53 reactivation with induction of massive apoptosis-1” (PRIMA-1) is a cyclic ketone from the family of quinuclidines with a relevant antitumor activity in several types of cancer. Functionally, PRIMA-1 restores mutp53 to a WT functional conformation, thereby re-establishing WTp53 transcriptional activity (Bykov et al., 2002; Zatloukalová et al., 2018). To reactivate mutp53, PRIMA-1 and PRIMA-1^MET^ (APR246, eprenetapopt) are first converted to a common active metabolite, 2-methylene-3-quinuclidinone (MQ). MQ acts through the Michael addition reaction, in which it covalently reacts with thiol groups of cysteine residues in the DBD (residues 94–312) of mutp53, restoring WTp53 functions (Lambert et al., 2009; Duffy et al., 2017; Perdrix et al., 2017).

The activities of PRIMA-1 and PRIMA-1^MET^ have been widely described in the literature, demonstrating the decreased proliferation and induction of apoptosis in tumor cells through the activation of various p53 targets (Lambert et al., 2009; Zhu et al., 2019; Ferretti et al., 2022). The combination of PRIMA-1^MET^ with cisplatin and 5-fluorouracil has been used in a phase II study concluded in 2020 with esophageal cancer patients (ClinicalTrials.gov Identifier: NCT02999893). For myelodysplastic syndrome and acute myeloid leukemia, 87% of the overall remission rate has been reported with the combination of PRIMA-1^MET^ and azacitidine (Sallman, 2020; Menichini et al., 2021). However, there are no clinical studies targeting p53 aggregates. Our group previously demonstrated that PRIMA-1 prevents p53 aggregation and inhibits the prion-like effects of mutp53 in breast and ovarian cancer cells by reducing the levels of intracellular amyloid oligomers of R280K and R248Q p53 mutants along with the reactivation and restoration of WTp53 activity (Rangel et al., 2019).

Several studies have demonstrated the potential of mutp53 amyloid aggregation as an actionable pharmacological target (Herzog et al., 2015; Soragni et al., 2016; Rangel et al., 2019; Palanikumar et al., 2021); a detailed review is given by Ferretti et al. (2022). However, no studies have addressed this possibility in the context of HCC. In this study, we demonstrate the amyloid character of p53 mutants in an HCC cell model using cell lines Huh-7 and PLC/PRF/5, from the *in silico* analysis of the p53 mutants present in the cell lines to a 3D-cell culture model. We selected one of these cell lines (Huh-7) to also describe the modulation of Y220C mutp53 aggregation by PRIMA-1. We demonstrate the inhibitory effects of PRIMA-1 on mutp53 GoF-related cancer cell properties, including cell migration, colony formation, and spheroid formation. Furthermore, we show that WT function can be restored *via* p53-mediated apoptosis and the induction of canonical p53 target genes. Finally, we show that PRIMA-1 synergizes with cisplatin in mutp53 HCC cells, supporting the premise that PRIMA-1 may enhance the response of a subset of HCC patients undergoing cisplatin therapy. Taken together, these results highlight the importance of studying p53 amyloid aggregation as a novel therapeutic approach for the treatment of HCC.

## Materials and methods

### Characterization of aggregation propensity

The WTp53 sequence (P04637) was collected from the UniProt website (https://www.uniprot.org/), and aggregation propensity of the point mutants Y220C, R249S, R248Q, and R280K was evaluated with different prediction algorithms, namely, AGGRESCAN (http://bioinf.uab.es/aggrescan/), TANGO (http://tango.crg.es/), Waltz (https://waltz.switchlab.org/), and ZipperDB (https://services.mbi.ucla.edu/zipperdb/), respectively, according to each algorithm’s recommendations.

### Cell culture

Hep3B (p53 null), HepG2 (WTp53), Huh-7 (Y220C mutant p53), and PLC/PRF/5 (R249S mutant p53) cell lines were purchased from the Rio de Janeiro Cell Bank (BCRJ, RJ, Brazil). All HCC cells were grown in Eagle’s Minimum Essential Medium (EMEM) with 10% fetal bovine serum, 1% L-glutamine, and 0.1% gentamicin (10 mg/mL). Cells were maintained at 37°C in a humidified atmosphere containing 5% CO_2_.

### Cell lysate preparation and Western blotting

Cells cultured in 75 cm^2^ flasks were washed three times with PBS and lysed with liquid nitrogen in a lysis buffer (10 mM Tris-HCl pH 7.5, 150 mM NaCl, 1 mM EDTA, and 1% Triton X-100) with a protease inhibitor cocktail (Sigma-Aldrich, USA), followed by centrifugation at 1,000 ×*g* for 5 min. The protein content was quantified by Lowry et al*.* (1951) and stored at −80°C (Lowry et al., 1951). An amount of 150 μg of the cell lysates was run on 12.5% sodium dodecyl sulfate–polyacrylamide gel electrophoresis (SDS-PAGE) and transferred to polyvinylidene difluoride low-fluorescence membranes (Millipore). The membranes were blocked with LI-COR blocking buffer for 1 h at 4°C and incubated overnight with anti-p53 antibody (1:10,000) (DO-1, Santa Cruz Biotechnology), anti-p21 antibody (Cell Signaling), or anti-MDM2 antibody (Cell Signaling), and then followed by IR-Dye® 800CW goat anti-mouse antibody (LI-COR) (1:15,000) for 1 h at room temperature. β-Actin was used as a loading control (Sigma-Aldrich). Signal detection was carried out using the Odyssey detection system (LI-COR), according to the manufacturer’s instructions. The quantification of band intensity was performed with ImageJ software (version 1.43r, National Institutes of Health) and normalized against HepG2, a WT-expressing cell line.

### Dot-blot assay

For dot-blot assays, 2.5 μg of protein lysates were placed onto a nitrocellulose membrane in a final volume of 2 μL. The membrane was blocked with LI-COR blocking buffer for 1 h at 4°C and incubated overnight at 4°C with the primary antibody A11 at a 1:5,000 dilution (Millipore). The membranes were washed five times with TBS-T, incubated with IR-Dye® 800CW goat anti-rabbit secondary antibody (1:10,000) at room temperature for 1 hour, and washed three times again with TBS-T and twice with TBS. The analysis was performed using the Odyssey detection system (LI-COR) and BSA and MDA-MB-231 cell lysates (which expresses the p53 mutant R280K), which were used as negative and positive controls, respectively. The analysis of the results was performed by densitometry with ImageJ software (version 1.43r, National Institutes of Health) and was normalized against HepG2, which was set as 1 (Rangel et al., 2019).

### Immunofluorescence colocalization assays

For the immunofluorescence colocalization assay, 10^5^ cells/wells were seeded in 24-well plates and allowed to adhere overnight. The cells were then washed with PBS, fixed with a methanol/acetone solution (1:1) for 10 min at −20°C, and incubated for 2 h with 100 µL of primary antibodies at 37°C with 5% CO_2_. The following antibodies were used: anti-p53 DO-1 (Santa Cruz Biotechnology, USA) (1:200) and A11 anti-amyloid oligomers (Millipore) (1:1,000) in blocking buffer (10% glycerol, 0.2% Tween 20, and 2% BSA in PBS). Subsequently, the cells were incubated with Hoechst 33258 (ThermoFisher, USA) (1:1,000), Alexa 568-conjugated goat anti-mouse, and Alexa 647-conjugated goat anti-rabbit (ThermoFisher, USA) secondary antibodies (1:2,000) for 1 h at room temperature, protected from light. After washing with PBS, coverslips were mounted with ProLong Diamond (ThermoFisher, USA) and analyzed by confocal microscopy (Leica TCS SPE confocal microscope, Carl Zeiss Inc.).

### Immunoprecipitation (IP) assays

Cell lysate (500 µg) was incubated with anti-p53 (DO-1) antibody (1:10,000) (Santa Cruz Biotechnology) or anti-amyloid oligomers (A11, Millipore, USA) in PBS for 1 h at 4°C. Then, 20 µL of protein A/G PLUS-Agarose (Santa Cruz Biotechnology, USA) was added and incubated overnight at 4°C. The next day, the samples were washed four times with PBS and centrifuged at 1,000 × *g* for 5 min. For Western blotting of the IPs (IP.WB), the samples were solubilized in 1X sample buffer (glycerol, 10% SDS, 0.5M TRIS pH 6.8, and beta-mercaptoethanol) and separated by SDS-PAGE (12,5%). The same procedures described in the Western blotting section were performed. In the membrane containing the samples immunoprecipitated with A11, antibody DO-1 (mouse) and secondary anti-mouse antibody IRDye® 800CW (LI-COR, USA) were used (Ferretti et al., 2019). For the dot-blot assay (IP.DB), 17 µL of 0.2 M glycine buffer pH 2.6 (in distilled water) was added to the samples and they were incubated for 10 min under agitation and centrifuged at 800 × *g* for 2 min at 4°C. An equal volume of Tris-HCl pH 8.0 buffer was added, and the samples were quantified by the Lowry method. Then, the dot-blot assay was performed using the anti-amyloid oligomer antibody (A11, Millipore, USA) (Navalkar et al., 2021).

### Protein purification and seeding of WTp53C aggregation

The expression and purification of WTp53C (residues 94-312) were performed, as described by Rangel et al. (2019). Protein samples were stored in 150 mM NaCl, 5 mM dithiothreitol (DTT), 50 mM Tris-HCl (pH 7.2), and 5% glycerol (v/v) at 80°C. The potential to induce the aggregation of WTp53C by the aggregated mutant protein present in Huh-7 or PLC/PRF/5 cell lysates was investigated by seeding assays. WTp53C (5 µM) was incubated with 25 µM of thioflavin T (ThT) with or without 3 μg/mL of Huh-7 or PLC/PRF/5 cell lysates for 5 min. Then, aggregation kinetics experiments were performed for 2 h at 37°C in an ISS-PC1 spectrofluorometer (ISS, Champaign, USA) with a wavelength of 440 and 482 nm in excitation and emission, respectively (Ishimaru et al., 2003; Rangel et al., 2019). The same procedure was performed using the cell lysates only, as a control.

### Cytotoxicity assay

Cells were seeded in 96-well plates and allowed to adhere until cells reached 70%–90% confluency. The cells were then treated with 100 μL of PRIMA-1 and PRIMA-1^MET^ in serial dilutions from 3.125 to 200 µM. The next day, 30 µL of MTT (0.5 mg/mL) in PBS was added, and the plates were incubated for 2 h. The formazan crystals were solubilized in 100 µL of DMSO, and the plates were analyzed with a SpectraMax Paradigm multi-mode microplate reader (Molecular Devices) at 570 and 650 nm.

### Reversibility assay

Huh-7 cells were seeded in 96-well plates and allowed to adhere until 70%–90% confluency was reached prior to the start of the assay. The cells were treated with 25, 50, and 100 µM of PRIMA-1 for the following: (1) 24 h, (2) 24 h with the cell culture medium being removed, and fresh EMEM being added and cells cultivated for a further 24 h (24 + 24 h), and (3) for 48 h. Cell viability was measured using the MTT assay, as described previously.

### Total RNA extraction and cDNA synthesis

Total RNA was extracted from Huh-7 cells’ monolayer in six-well plates at 80%–90% confluency using TRIzol (Invitrogen), according to the manufacturer’s recommendations. Total RNA quantification and purity were assessed in a NanoDrop® ND2000 microvolume spectrophotometer (ThermoFisher), at 260, 280, and 320 nm. Total RNA (1 μg) was treated with DNAse I (Sigma-Aldrich), according to the manufacturer’s recommendations. Reverse transcription was performed from DNAse-treated RNA using a Superscript III First Strand cDNA Synthesis Kit (Invitrogen, USA), according to the manufacturer’s instructions. cDNAs were diluted to 1:10 before use.

### Analysis of gene expression by RT-qPCR

For gene expression assessment of p53, MDM2, NOXA, and p21 targets, real-time quantitative PCR (RT-qPCR) was carried out in a 10 μL reaction containing 5 μL of [2X] Power SYBR Green (Applied Biosystems), 300 nM of forward and reverse primers (see as follows), 2 μL cDNA, and nuclease-free water to reach 10 μL. Real-time PCR reactions were carried out on a ViiA 7 Real-Time PCR System (Applied Biosystems) using the following cycling conditions: 10 min at 95°C, followed by 40 cycles of 15 s at 95°C, and 60 s at 62°C. Fluorescence was observed after each cycle at the annealing/extension step. All samples were run in duplicates, and the threshold was set at 0.02 for all targets. The results were analyzed using ExpressionSuite v1.0.3 (Applied Biosystems, USA), GAPDH and 18S targets were selected as endogenous controls, and gene expression was estimated using the ΔΔCt method (Livak and Schmittgen, 2001). Relative quantification was estimated using the control (untreated) sample as the calibrator. The primers designed for this study were p53 Fw: 5′-TGA​CAC​GCT​TCC​CTG​GAT​TG-3′ and p53 Rv: 5′-TTT​TCA​GGA​AGT​AGT​TTC​CAT​AGG​T-3′; MDM2 Fw: 5′-AGG​AGA​TTT​GTT​TGG​CGT​GC-3′ and MDM2 Rv: 5′-TGA​GTC​CGA​TGA​TTC​CTG​CTG-3′; NOXA Fw: 5′-CGG​AGA​TGC​CTG​GGA​AGA​AG-3′ and NOXA Rv: 5′-ACT​CGA​CTT​CCA​GCT​CTG​CT-3′; and p21 Fw: 5′-AGT​CAG​TTC​CTT​GTG​GAG​CC-3′ and p21 Rv: 5′-GAC​ATG​GCG​CCT​CCT​CTG-3′. To the reference genes, primers GAPDH Fw: 5′-ATG​TTC​GTC​ATG​GGT​GTG​AA-3′ and GAPDH Rv: 5′-GGT​GCT​AAG​CAG​TTG​GTG​GT-3′ and 18S Fw: 5′-CAG​CCA​CCC​GAG​ATT​GAG​CA-3′ and 18S Rv: 5′-TAG​TAG​CGA​CGG​GCG​GTG​TG-3′ were used (Rocha et al., 2014).

### Migration assay

For migration assays, cells were seeded in a 24-well plate until 80% confluency was reached. Cell monolayers were scratched with a sterile p200 tip, washed with PBS, and treated with 25, 50, and 100 µM PRIMA-1 containing mitomycin C (0.5 μg/mL) for the inhibition of cell proliferation. Images were obtained on the day of treatment and after 48 h with an EVOS brightfield microscope (EVOS M5000 Cell Imaging System, Life Technologies), and measurements were performed using ImageJ software (version 1.43r, National Institutes of Health).

### Colony formation assay

Huh-7 cells were seeded in a 24-well plate until 80% confluency was reached. Then, the cells were treated with PRIMA-1 (25, 50, and 100 µM). After 24 h, viable cells were counted with trypan blue and 500 cells/well were added to a six-well plate (final volume of 2 mL/well). After 7 days, colonies were stained with crystal violet solution (0.5% and 25% methanol in water) and washed abundantly with distilled water.

### 3D-cell culture assays

3D-cell cultures were performed, as previously described (Friedrich et al., 2009). Briefly, in a 96-well plate, 50 µL of agarose 1% was added per well. After agarose solidification, 100 µL of culture medium containing 4 × 10^3^ cells was plated and treated with 50 and 100 µM of PRIMA-1. Then, the plate was centrifuged at 400 × *g* for 10 min. For the spheroid formation inhibition assay, treatment was performed concomitantly with spheroid establishment: PRIMA-1 was added and diluted in culture medium while plating and incubated for 72 h at 37°C in 5% CO_2_. Then, spheroid formation and cell viability were assessed. To evaluate PRIMA-1 effects on the formed spheroids, the same procedure was performed without PRIMA-1 addition, and only after 72 h, the formed spheroids were treated with PRIMA-1 (50 and 100 µM). After 48 h, spheroid images were obtained with an EVOS brightfield microscope (EVOS M5000 Cell Imaging System, Life Technologies).

### Acid phosphatase (APH) assay

The viability of the spheroids was evaluated by the acid phosphatase (APH) assay. A measure of 100 μL of the medium from 3D-cell cultures was removed and 100 µL of APH buffer was added, containing 2 mg/mL of p-nitrophenyl phosphate (PNPP) and 0.1% Triton X in 0.1 M citric acid. After 2 h, 10 µL 1M NaOH was added and absorbance was read at 405 nm and 630 nm (Ivanov et al., 2014).

### Annexin V-FITC/propidium iodide apoptosis detection

Cells (10^6^) were seeded in a 24-well plate until ∼80% confluency was reached. Cells were treated with PRIMA-1 (25 and 50 µM), and cisplatin (200 µM) was used as a positive control for apoptosis. After 24 h of treatment, the cells were centrifuged at 460 × *g* for 5 min, washed twice with PBS, and treated using an Apoptosis Detection Kit (ThermoFisher, USA), according to the manufacturer’s instructions. The samples were then analyzed in a Countess II FL Automated Cell Counter (ThermoFisher, USA).

### PRIMA-1 and cisplatin combination assay

HepG2 and Huh-7 cells were simultaneously treated with PRIMA-1 (25, 50, and 100 µM) and cisplatin (from 50 to 1,600 µM), as described previously for the cytotoxicity assays. After 24 h, the MTT assay was performed. The combination index was evaluated with CompuSyn software (http://www.combosyn.com/) using the Chou–Talalay combined index method. Synergistic, additive, and antagonistic effects are shown by CI < 1, CI = 1, and CI > 1, respectively (Chou, 2006; Zhang et al., 2020).

### Statistical analysis

Statistical analysis was performed using the Prism 8.0 program (GraphPad Software, USA). Data were analyzed by the Student’s t-test, and *p* < 0.05 values were considered statistically significant. For gene expression analysis by RT-qPCR, a normality test was carried out by performing the Shapiro–Wilk test, followed by Student’s t-test with SigmaPlot for Windows version 14.0 (Systat Software, Inc.), using the ΔCt values. The fold change results were expressed as means and standard deviations. Differences were considered significant if *p* < 0.05, as described in each figure legend.

## Results and discussion

### *In silico* analysis of WTp53 and mutp53 reveals similar aggregation tendencies

We performed *in silico* analysis to generate comparisons between the aggregation potential of p53 sequences, wild-type, and mutants, by using three different tools to analyze these sequences: AGGRESCAN, TANGO, WALTZ, and ZipperDB. AGGRESCAN is a web-based software application that can predict aggregation-prone sequences in proteins and compare the effect of mutations or different protein sequences (Conchillo-Solé et al., 2007). TANGO is an algorithm designed for the prediction of regions able to nucleate aggregation and also the effects of mutations and environmental conditions on this process (Rousseau et al., 2006). WALTZ is an algorithm designed for the prediction of amylogenic regions in protein sequences based on experimental data (Oliveberg, 2010). Finally, ZipperDB is a method built for the prediction of sequences that can form complementary ß-sheets with a high fibrillation propensity (Goldschmidt et al., 2010). The sequences refer to the mutations found in the cell lines used in this work: Y220C (Huh-7) and R249S (PLC/PRF/5). The aggregation potential of mutants R248Q and R280K was also included since they were used in previous studies from our group (Ano Bom et al., 2012; Rangel et al., 2019).

**FIGURE 1:**
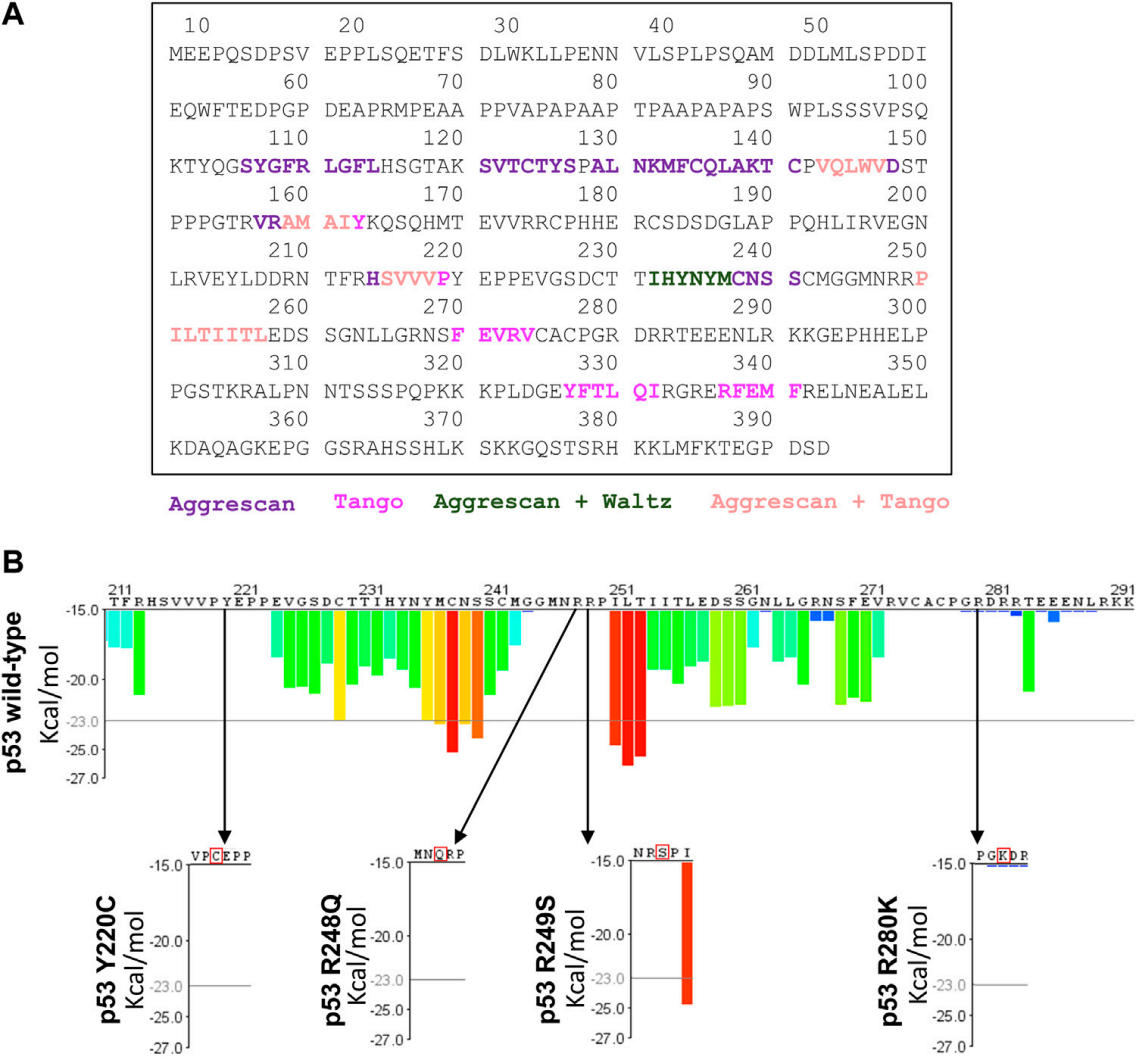
Aggregation prediction for p53 mutants using different algorythms. Primary sequence of p53 and aggregation hotspots colored for each aggregation prediction parameter. **(A)** Mutant p53 sequences Y220C, R249S, R248Q, and R280K with the highest aggregation-prone sequences colored in purple (AGGRESCAN), violet (TANGO), dark green (AGGRESCAN + WALTZ), and light pink (AGGRESCAN + Tango). **(B)** Representation of the Rosetta energy for the aggregation-prone segments in the p53 sequence predicted by ZipperDB.

Several parts of the WTp53 primary sequence depict tendencies to induce aggregation or fibrillation that vary according to the prediction algorithm and sometimes overlap. The exact same results were found for missense mutations Y220C, R249S, R248Q, and R280K (Figure 1A), in which AGGRESCAN indicated an aggregation propensity in amino acid sequences 106–114 (SYGFRLGFL), 121–127 (SVTCTYS), 129–141 (ALNKMFCQLAKTC), 143–148 (VQLWVD), 157–162 (VRAMAI), 214–218 (HSVVV), 232–241 (IHYNYMCNSS), and 251–257 (ILTIITL); TANGO indicated a tendency to enrichment in ß-sheets for fragments 143–147 (VQLWV), 159–163 (AMAIY), 215–219 (SVVVP), 250–257 (PILTIITL), 270–274 (FEVRV), 327–332 (YFTLQI), and 337–341 (RFEMF); Waltz showed a tendency toward the formation of amyloid sequences at 232–237 (IHYNYM); and the ZipperDB analysis for WTp53 and mutants also showed equivalent results, indicating a region with the highest propensity to aggregate between positions 252 and 258, reported previously by Soragni et al. (2016). It is interesting to notice that positions 248 and 249 neighbor this segment, but neither R248Q nor R249S mutations alter the high tendency to form ß-strands attributed to this region (Figure 1B).

These results are consistent with previous studies (Rangel et al., 2014; Soragni et al., 2016). Particularly, the sequences 232–237 (IHYNYM) and 327–332 (YFTLQI), found in WTp53 and in Y220C, R249S, R248Q, and R280K mutants are 93.3% (Waltz) and 18.5% (TANGO) prone to aggregation, respectively. In particular, the sequence 251–257 (ILTIITL) on the S9 ß-strand is often mentioned because it has a high propensity for aggregation. It is 95.5% (TANGO) likely to have ß-strands that cause protein aggregation, which favors therapeutic resistance and tumor development (Ghosh et al., 2014). It is worth noting that there are regions protected against proteolytic degradation in p53, the main sequences being 249–267 (S9), 268–282 (S10H2), 102–120 (S1L1), and 182–213 (H1S5S6) (Wang and Fersht, 2017). We observed that most of these protected regions have a sequence with a propensity for intercalated aggregation, which may suggest that these regions prevent proteolytic degradation, favoring the formation of aggregates in the most prone regions and leading to the accumulation of aggregates in cells (Wang and Fersht, 2017). Interestingly, the 10 cysteine residues present along the p53 sequence are all located within the DBD (residues 94–312): Cys124, 135, 141, 176, 182, 229, 238, 242, 275, and 277. Of these, six are either contained by or closely proximal to the sequences featured by the amyloid aggregation predictors used here, and for the Y220C mutant, an extra cysteine is included in this group. The cysteine residues present along the p53 sequence are critical for the effects promoted by PRIMA-1 (Lambert et al., 2009) with a special emphasis on Cys124 and Cys277, which have shown to be the most reactive cysteine residues and to act as a prime-binding target for MQ (Zhang et al., 2018). Y220C is a mutant that forms a peculiar cavity with a druggable potential. Furthermore, assays with the protein core domain (DBD) have shown its aggregation potential (Wilcken et al., 2012).

### p53 mutants Y220C and R249S are found in the amyloid state in HCC cell lines but with different cellular localizations

**FIGURE 2:**
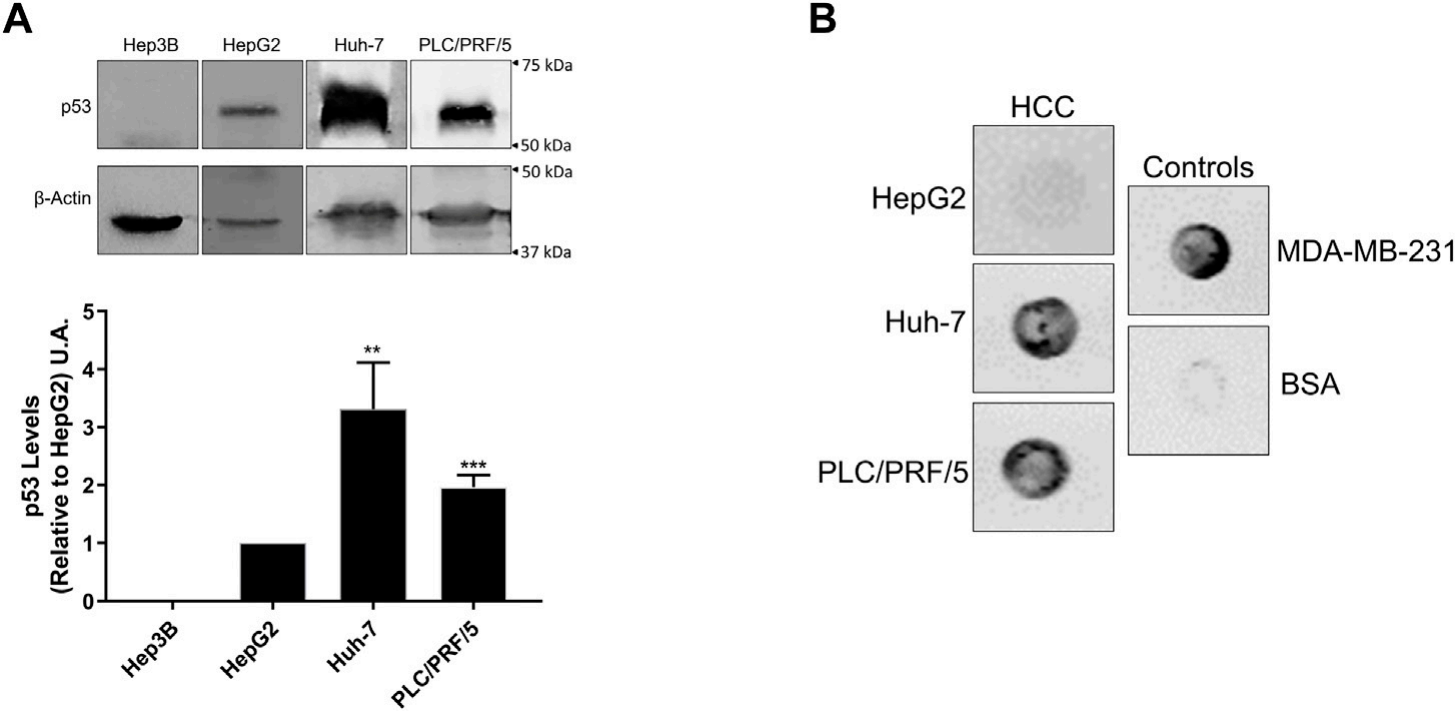
p53 and amyloid oligomer levels in hepatocellular carcinoma cell lines. **(A)** Western blot of HCC cell lines (upper panel) and quantification of p53 levels relative to HepG2 (lower panel). Huh-7 and PLC/PRF/5 show higher p53 levels than HepG2. The mean ± error analysis was calculated from three independent experiments, where **p < 0.005 and ***p < 0.001. **(B)** Dot-blot on nitrocellulose membrane incubation with the A11 antibody, showing amyloid oligomer staining for both HCC cell lines and MDA-MB-231 cells.

One of our aims was to establish an HCC model for p53 aggregation studies. Toward this goal, multiple HCC cell lines were compared with respect to their p53 expression, taking into account the p53 status for each cell line (p53 null, WT, or mutant). Our results show that Huh-7 and PLC/PRF/5 (both expressing mutp53) present higher protein levels when compared to HepG2, which express WTp53. Huh-7 displays an accumulation of ∼3.3 times, and PLC/PRF/5, ∼2 times higher p53 expression than HepG2. Hep3B is a p53 null cell line and did not show any expression of p53 (Figure 2A). Huh-7 shows higher p53 accumulation than the other cell lines (Iwao and Shidoji, 2014), as expected, due to its increased stability and the longer half-life of the protein (Bressac et al., 1990).

To test the hypothesis that p53-mutant HCC leads to amyloid formation, we performed dot-blot assays with the anti-amyloid oligomer antibody A11 on multiple HCC cell lines (Figure 2B). Bovine serum albumin (BSA), which does not form amyloid oligomers under normal conditions, was used as a negative control. MDA-MB-231, a breast cancer cell line carrying the R280K p53 mutation that was previously shown by our group to form amyloid oligomers, was used as a positive control (Ano Bom et al., 2012; Rangel et al., 2019). We observed for both HCC mutp53 cell lines a large amount of amyloid oligomers for both HCC mutp53 cell lines, as observed in MDA-MB-231 cells.

**FIGURE 3:**
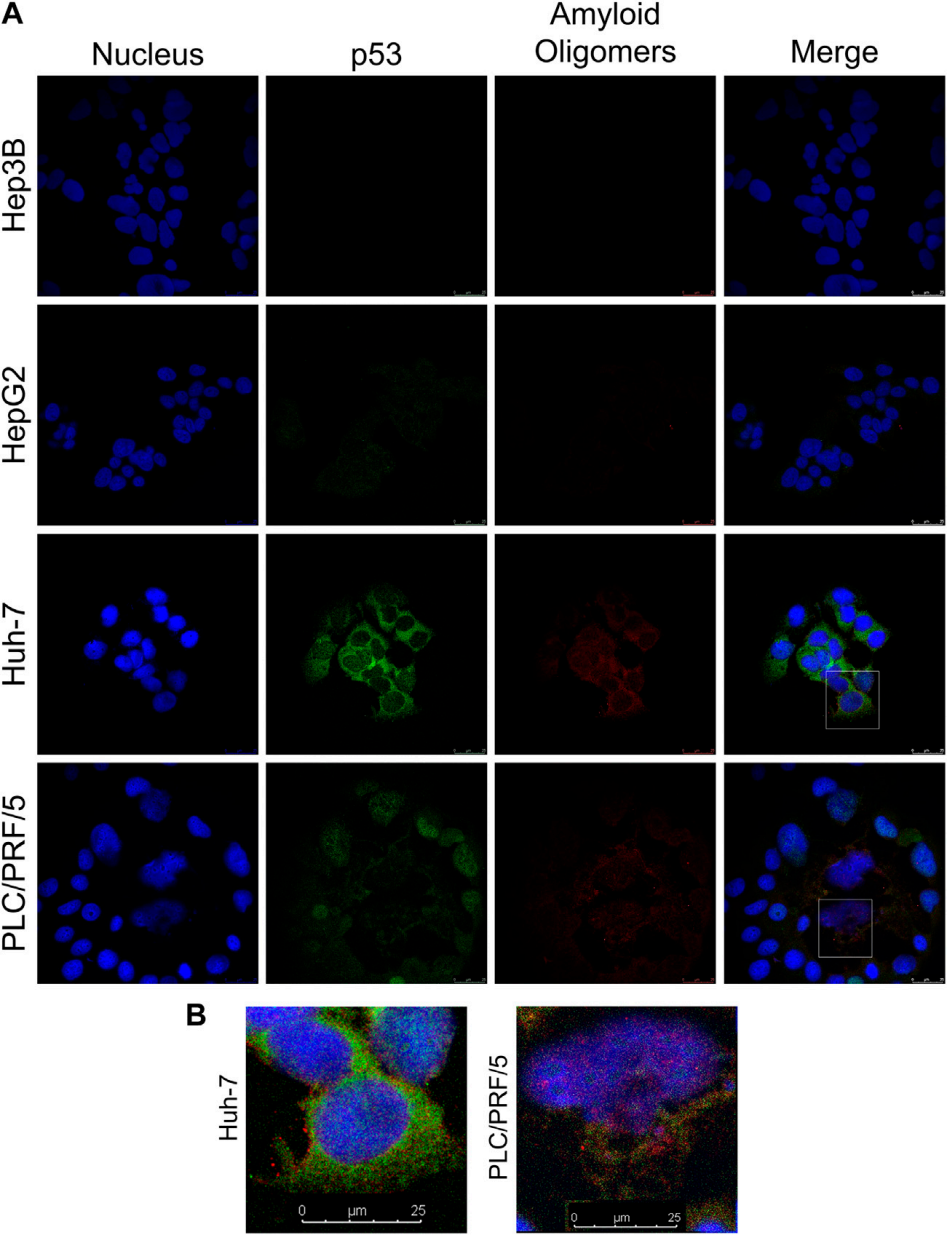
Intracellular colocalization of p53 amyloid oligomers in HCC cell lines. **(A)** Cells were labeled with anti-p53 (DO-1) and anti-oligomer (A11) antibodies, and the nuclei were stained with Hoechst. **(B)** Closer view of the highlighted regions in panel A of Huh-7 and PLC/PRF/5 with p53 amyloid oligomers (white arrow heads). Scale bars: 25 µm.

To determine whether the amyloid aggregates present in HCC cells are related to p53 and their subcellular localization, fluorescence confocal microscopy was used (Figures 3A, B). Hep3B did not show any significant labeling for both antibodies, as expected for a cell that does not express p53 (Vollmer et al., 1999). HepG2 demonstrated weak staining for both p53 and amyloid oligomers. In Huh-7, p53 and amyloid oligomers were more prominently distributed in the cytoplasm, while in PLC/PRF/5, a concentration of p53 was also observed in the nucleus of some of the cells. Merged images of Huh-7 cells suggest amyloid oligomers and p53 colocalization are present more in the cytoplasm, while in PLC/PRF/5, the aggregates are found in both the cytoplasm and the nucleus. These differences can be observed in more detail in Figure 3B, in which the amplifications of the selected areas of Huh-7 and PLC/PRF/5 from Figure 3A are shown.

Other studies have analyzed the presence of p53 aggregates in tumors and their location within the cell. Colocalization between p53 and amyloid oligomers has been reported in breast cancer cell lines expressing the R248Q mutant, and tumor aggressiveness appears to be strongly correlated with p53 aggregation (Ano Bom et al., 2012). In breast cancer patient samples, colocalization is more predominant in the cytoplasm for different p53 mutations (Levy et al., 2011). p53 aggregates are also found in different cells and with different p53 mutants in the cytoplasm (Xu et al., 2011), including NUGC3 gastric adenocarcinoma cells, which harbor the Y220C mutation (Miller et al., 2019). The presence of p53 and amyloid oligomers in liver tissues of rats, healthy or with HCC, has also been studied. In healthy tissue, p53 staining is very low and there is almost no staining for amyloid oligomers. In contrast, in hepatocellular carcinoma tissue, strong staining is observed for p53 and amyloid oligomers that colocalize in the cell cytoplasm (Ghosh et al., 2017). Our results suggest that the Y220C (Huh-7) mutation leads to the formation of p53 amyloid oligomers in mutant hepatocellular carcinoma cell lines that are no longer able to enter the nucleus or be degraded, thus accumulating in the cytoplasm, while the R249S mutant (PLC/PRF/5) is also present in the nucleus, which may impact p53 function (Baptiste and Prives, 2004; Lavin and Gueven, 2006; Green and Kroemer, 2009; Ghosh et al., 2014).

**FIGURE 4:**
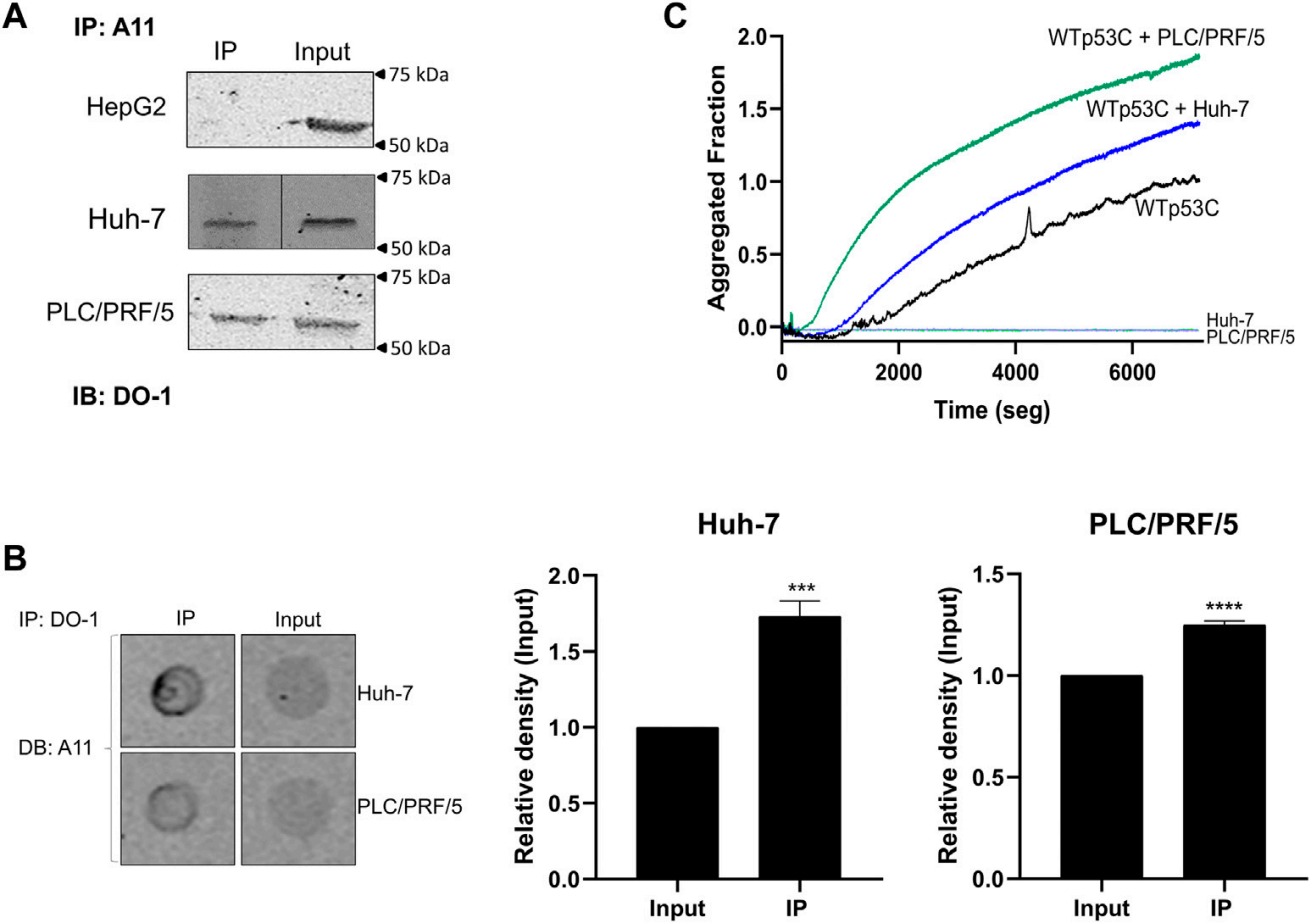
Amyloid-state and prion-like effect of mutant p53 HCC cell lines. **(A)** IP with A11, followed by immunoblotting (IB) with DO-1. **(B)** IP of amyloid oligomers with p53 with DO-1, followed by dot-blot (DB) assay with A11. The difference was statistically significant in IP.DB quantification (****p < 0.0001, *n* = 3). Input: cell lysate. **(C)** WTp53C aggregation alone (black line) or seeded by mutant p53 HCC cell line lysates: PLC/PRF/5 (green line) and Huh-7 (blue line). The light green line corresponds to the cell lysate alone used as the seed. Lysates (3 μg/mL) were used to induce WTp53C (5 µM) aggregation in a solution containing 50 mM Tris (pH 7.2), 150 mM NaCl, 5 mM DTT, and 5% glycerol, followed by ThT (25 µM) fluorescence (440 nm excitation and 482 nm emission) at 37°C. Aggregated fraction = (Fobs-FI)/(FF-FI), where F is the ThT fluorescence emission, Fobs is the observed fluorescence emission, FI is the initial fluorescence, and FF is the final fluorescence. Each of the images shows an experiment representative of three independent experiments.

To further confirm the amyloid oligomer status of mutp53 in HCC cell lines, we used two different immunoprecipitation (IP) approaches: IP using A11 and Western blotting with DO-1 (which recognizes a linear epitope) (IP.WB) and IP using DO-1, followed by a dot-blot assay with A11 (which recognizes a conformational epitope) (IP.DB). The anti-amyloid oligomer antibody A11 is not specific for p53 (Glabe, 2004). However, in the IP.WB assay, amyloid oligomers were immunoprecipitated and their p53 content was detected by Western blotting. Conversely, in the IP.DB assay, total p53 was immunoprecipitated and its amyloid content was measured by dot-blot assay, making these experiments more specific for the amyloid fraction of p53 present in the cell lysate. We observed the capture of amyloid oligomers from Huh-7 and PLC/PRF/5 mutp53 cell lines by immunoprecipitation, detected by Western blotting using an anti-p53 antibody (Figures 4A, B). Unsurprisingly, HepG2 cells did not present any amyloid-state p53, given their WTp53 status.

Our group has previously performed experiments using the R248Q mutant or MDA-MB-231 protein lysates to demonstrate a prion-like effect of the aggregated mutp53 on WTp53C (DBD) aggregation kinetics (Ano Bom et al., 2012; Rangel et al., 2019). We observe evidence of a prion-like behavior for the mutp53 HCC cell lines, as shown in Figure 4C, in which the lysates of Huh-7 and PLC/PRF/5 act as seeds for WTp53C, causing an increase and acceleration of its aggregation. Similar results were observed with the Y220C mutant by another group (Wang and Fersht, 2015). Given our findings of the amyloid properties presented by the mutp53 HCC cell lines used in this study, we decided to test the effects of a class of mutp53 reactivators, PRIMA-1 and PRIMA1-^MET^, on these cell lines.

### PRIMA-1 and PRIMA1-^MET^ reduce the viability of hepatocellular carcinoma cell lines with p53 mutations

**FIGURE 5:**
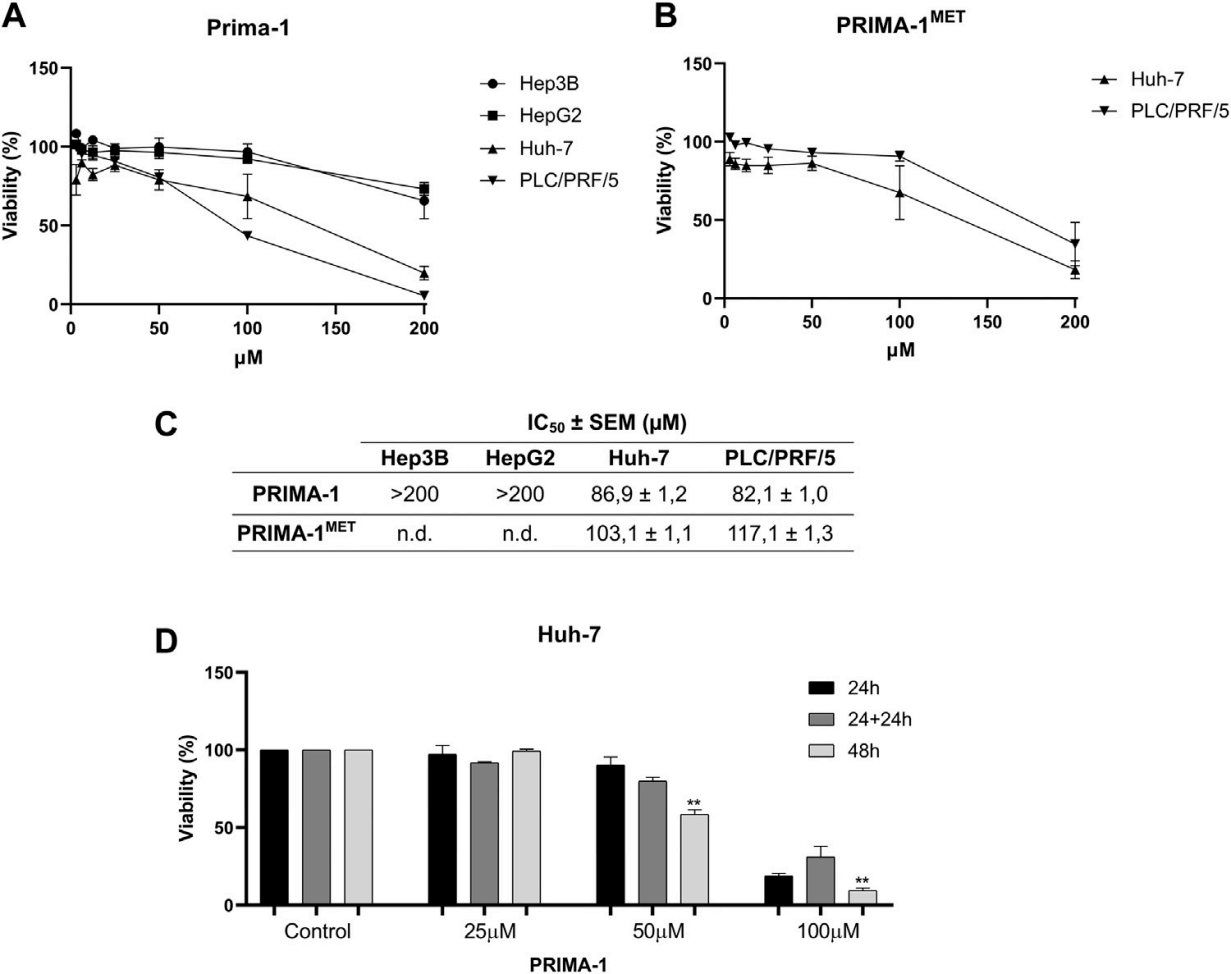
PRIMA-1 and PRIMA-1^MET^ preferentially inhibit HCC mutant p53 cell lines. Cells were treated with different concentrations of PRIMA-1 **(A)** and PRIMA-1^MET^ **(B)** for 24 h and analyzed by MTT. **(C)** IC_50_ values were obtained from the dose-response curves. **(D)** Reversibility assay of Huh-7 cells treated with 25, 50, and 100 µM PRIMA-1 for 24, 24 + 24, and 48 h, respectively. Statistical analysis in relation to 24 h treatment **p < 0.005). SEM: standard error. n.d. not detected.

Based on our characterization of the HCC model for p53 aggregation, we then decided to test its ability to be used for the screening of new compounds as inhibitors of p53 aggregation and p53 reactivation with apoptosis induction and/or cell chemosensitization by the reduction of mutp53 levels in the cell (Freed-Pastor and Prives, 2012). For this, we used PRIMA-1, a known inhibitor of mutp53 R280K and R248Q aggregation with reactivation (Rangel et al., 2019), and PRIMA-1^MET^, its methylated form. HCC cell lines were treated with PRIMA-1 (Figure 5A) and PRIMA-1^MET^ (Figure 5B) with increasing concentrations ranging from 1.56 to 200 μM. Mutp53 Huh-7 and PLC/PRF/5 cell lines presented the lowest IC_50_ values, with 86.9 ± 1.2 and 82.1 ± 1.0 µM, respectively (Figure 5C). When compared to PRIMA-1, treatment with PRIMA-1^MET^ (Figure 5C) produced higher IC_50_ values: 103.1 ± 1.1 µM for Huh-7 and 117.7 ± 1.3 µM for PLC/PRF/5. These data led us to choose PRIMA-1 to further characterize p53 amyloid aggregation inhibition and focuse on the cell line that we found to have higher amyloid p53 levels, Huh-7. These cells were also chosen based on the cytoplasmic localization of the p53 aggregates of Y220C, along with its peculiar structure and aggregation potential, as previously described (Wilcken et al., 2012), which are different from the mutants used in our previous study (Rangel et al., 2019).

We, then, wanted to confirm the reversibility of PRIMA-1 effects on this cell line (Figure 5D). Cell viability was reduced in a dose- and concentration-dependent manner (Figures 5A, C), which did not seem to be reversed by treatment withdrawal (Figure 5D). There was a significant reduction in cell viability to 58.5% in a 48-h treatment with 50 µM of PRIMA-1. With 100 µM of PRIMA-1, there was a reduction in cell viability at the time points tested, with viabilities of 18.9% (24 h), 32.1% (24 + 24 h), and 9.5% (48 h). The irreversibility of this effect was observed by the withdrawal of treatment after 24 h and fresh medium addition (24 + 24 h), and we observed no significant recovery of cell viability, in a similar result observed for the 48-h treatment (Figure 5D). This irreversibility is compatible with the mechanism described for PRIMA-1 with covalent binding of the active metabolite to cysteine residues present in p53 (Lambert et al., 2009).

PRIMA-1 has been shown to delay the growth of Hep3B cells ectopically expressing the R249S mutant of p53 in xenograft models using immunodeficient mice. This mutant was previously shown to exert a GoF by promoting cell survival (Shi et al., 2008). Gomes et al. (2019) found a minimum inhibitory concentration of approximately 40 µM for PRIMA-1^MET^ for Huh-7 (Y220C) through the sulforhodamine B assay (Gomes et al., 2019). In a separate work, cell viability was measured in Huh-6 (WTp53) and Huh-7 cell lines after treatment with PRIMA-1^MET^ with a concentration of 60 µM to reduce the viability of Huh-6 to 40% and Huh-7 to approximately 60%, showing the activity of this class of compounds in HCC cells (Bauer et al., 2016).

### PRIMA-1 decreases the levels of p53 amyloid oligomers in Huh-7 cells

**FIGURE 6:**
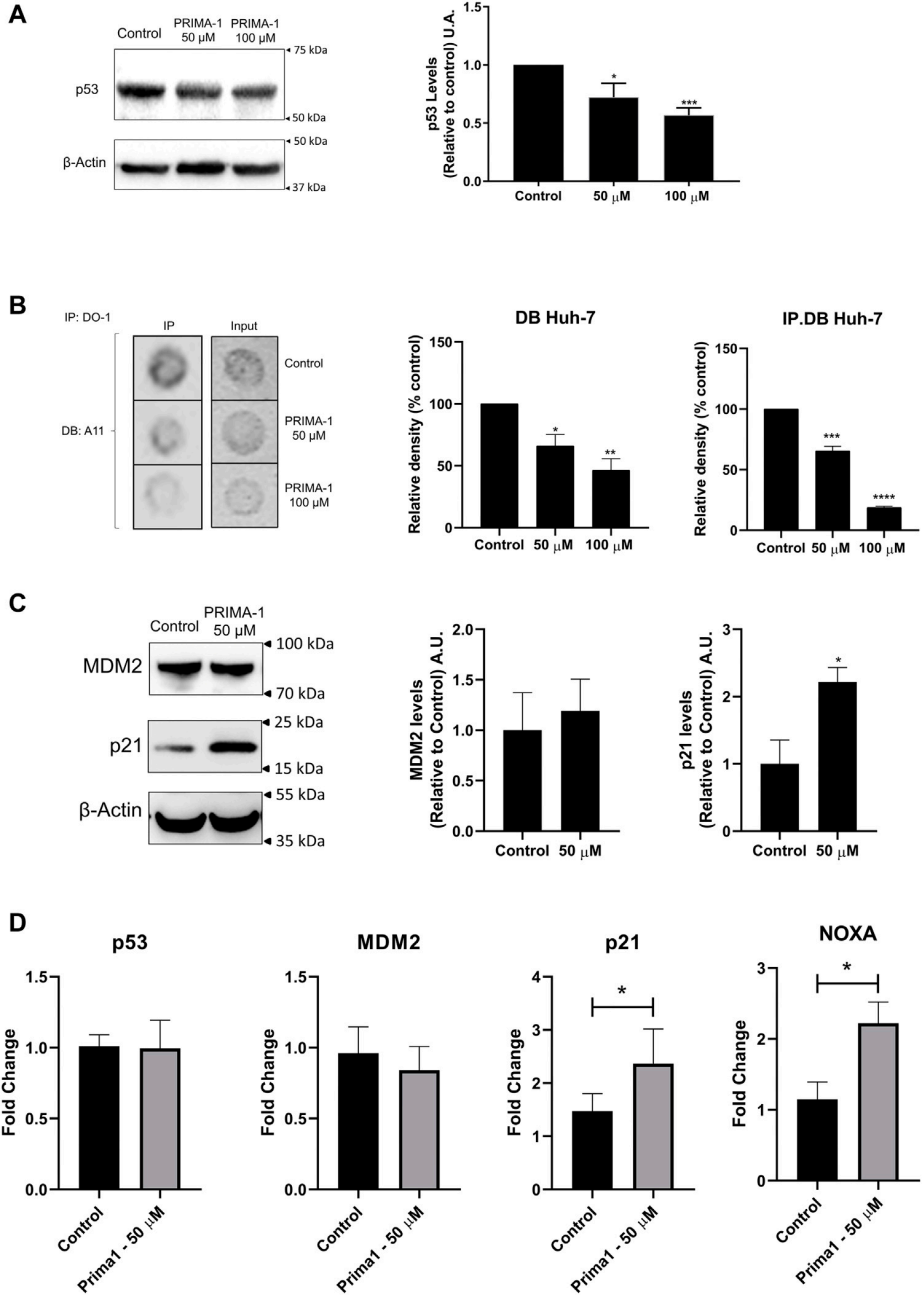
PRIMA-1 reduces amyloid p53 levels in Huh-7 cells. **(A)** p53 protein level of Huh-7 cells are reduced upon treatment with PRIMA-1. **(B)** Huh-7 cells treated with PRIMA-1 display lower levels of p53 amyloid oligomers detected by IP.DB using DO-1 and A11 antibodies. **(C)** p21 protein expression of Huh-7 cells is enhanced following treatment with PRIMA-1, while MDM2 levels are not altered. **(D)** RT-qPCR of p53 target genes show no changes in MDM2 and TP53 gene expression, while CDKN1A (p21) and PMAIP1 (NOXA) are activated. Quantification of p53, p21, and MDM2 levels was relative to control. Statistical analysis shows *p < 0.05; **p < 0.005; ***p < 0.001, and ****p < 0.0001.

Western blot analysis showed a significant reduction in p53 levels, following treatment with PRIMA-1 (Figure 6A), to 72.33% at 50 μM and 57% at 100 µM. A reduction of amyloid oligomers staining (Figure 6B) in response to incrementing PRIMA-1 concentrations was also observed, both in the dot-blot with the whole cell lysate (Figure 6B, input column) and in the IP.DB assays (Figure 6B, IP column). Quantification of the IP.DB indicated that the levels of amyloid oligomers in Huh-7 dropped to 66.1% at 50 μM and 46.5% at 100 µM. In IP.DB, the levels of p53 amyloid oligomers decreased to 65% (50 µM) and 18.8% (100 µM). In all the tested conditions, significant differences from the control and a dose-response profile were observed. However, the most specific approach used, IP.DB, demonstrated a more intense decay in amyloid p53 levels, when compared either with the total p53 content (WB values) or total amyloid content (DB). This marked decrease in amyloid p53 levels supports the premise that mutp53 is recovering its WT conformation and reactivating its transcriptional activity, although some degradation may be possible. To address this, we performed RT-qPCR analysis to evaluate whether canonical p53-target genes were reactivated by PRIMA-1. Figure 6D shows that the expression of p21 and NOXA increased following treatment with PRIMA-1. Although no effect was observed on gene expression with a reduction in protein levels, a possible degradation could be happening as well, but since MDM2 levels remained unaffected following PRIMA-1 treatment, another pathway or E3-ligase might be activated to promote p53 degradation. The increase in p21 protein levels is observed *via* Western blot analysis, even though MDM2 levels appear unaffected (Figure 6C). Taken together, we propose that, although total p53 levels are depleted, reduction in amyloid p53 levels (Figure 6B, IP.DB) is more prominent and accompanied by an increase in p53 transcriptional activity, suggesting the reactivation of p53 (Rangel et al., 2019; Ferretti et al., 2022).

### PRIMA-1 inhibits mutant p53 “gain-of-function” in Huh-7 cells

p53-related oncogenic GoFs are functions acquired by a cancer cell with a *TP53* mutation that are not related to the loss of WTp53 (Freed-Pastor and Prives, 2012; Alvarado-Ortiz et al., 2021). Many of the oncogenic GoFs attributed to p53 are related to its interaction with other members of the p53 family (p63 and p73), which, in turn, only occur when p53 is mutated (Li and Prives, 2007; Ferraiuolo et al., 2016). Part of the effects attributed to PRIMA-1 is associated to the inhibition of the interaction with p73, which, in turn, coaggregates with p53 (Xu et al., 2011; Kehrloesser et al., 2016). In addition, p53 aggregation has been related to the GoFs observed in cancer models (Xu et al., 2011; Pedrote et al., 2020). Among these GoFs are increased invasion, altered migration (Adorno et al., 2009; Muller et al., 2009), and drug resistance (Shetzer et al., 2014; Xu et al., 2020; Roszkowska et al., 2022). We sought to evaluate the functional effects of PRIMA-1 on GoF properties in Huh-7 cells.

**FIGURE 7:**
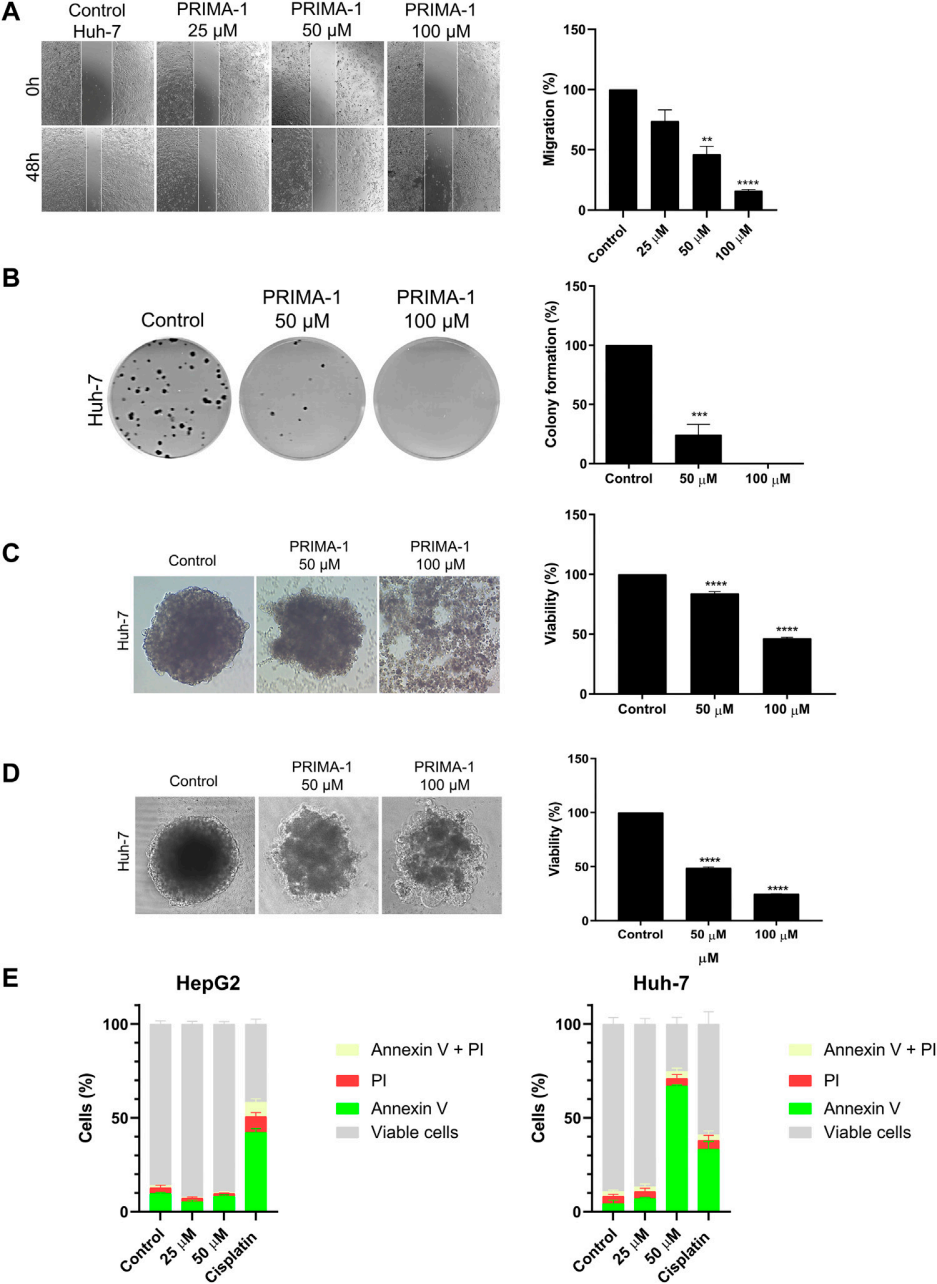
PRIMA-1 reduces GoF-related effects in HCC cells. **(A)** Migration of HepG2 and Huh-7 cells treated with PRIMA-1 (25, 50, and 100 µM). Values are expressed as a percentage of control migration. **(B)** Clonogenic assay of Huh-7 cells treated with PRIMA-1 (50 and 100 µM) and stained with crystal violet. **(C)** Huh-7 spheroids simultaneously prepared and treated with PRIMA-1. **(D)** Previously formed spheroids treated with PRIMA-1 at 50 and 100 µM. **(E)** Apoptosis detection using annexin V-FITC and propidium iodide (PI), with HepG2 and Huh-7 cells treated with PRIMA-1 (25 and 50 µM). Cisplatin (200 µM) was used as a positive control.

We found that PRIMA-1 (Figure 7A) suppressed cell migration in a concentration-dependent manner, and 48 h following treatment with PRIMA-1, Huh-7 cells migrated 73.8% (25 µM), 46.2% (50 µM), and 16% (100 µM) relative to the untreated control. A significant reduction of approximately 76% in colony formation was observed with 50 μM, and no colony formation was observed with 100 µM of PRIMA-1. When we compared these data with the reversibility assay (Figure 5D), these cells were unable to adhere and form colonies after 24 h without treatment, even though cells were viable in the 24 + 24 h condition. Thus, it may indicate that PRIMA-1 has an inhibitory effect on the motility of Huh-7 cells, in relation to their migration, adhesion, and colony formation.

The use of spheroids has been widely discussed due to its ability to mimic tumors *in vitro* and, in some cases, the tumor microenvironment, directly supporting the prospection of new drugs (Sutherland et al., 1981). In this assay, HCCs present a 3D architecture, unlike the monolayer assay (2D); thus, the cells are arranged in a way that is more similar to the tumor *in vivo*. Spheroids present a gradient of oxygen, nutrients, metabolites, and a proliferative and necrotic area that directly influence the therapeutic response (Mehta et al., 2012). Using the spheroid formation inhibition assay, Huh-7 spheroids were formed and concomitantly treated with 50 and 100 µM of PRIMA-1 (Figure 7C). Untreated control Huh-7 cells formed a compact spheroid without morphological changes. However, 72 h following treatment with 50 µM of PRIMA-1, the spheroids did not acquire the same shape as the control. When treated with 100 µM of the compound, the cells did not form spheroids at all. A separate assay with spheroids was carried out to evaluate the activity of PRIMA-1 in previously formed spheroids. Here, the spheroids were prepared and treated 48 h later with PRIMA-1. The effects were evaluated 48 h after treatment. We found that PRIMA-1 appears to induce a collapse of the spheroids, followed by their disruption at all tested concentrations (Figure 7D).

The acid phosphatase method was then used to assess the cell viability of spheroids. When spheroids were simultaneously prepared and treated, there was a significant reduction in viability to 84% with 50 µM PRIMA-1 (Figure 7C). At 100 μM, in addition to preventing the formation of spheroids, the treatment reduced cell viability by approximately 46%. Although with an altered shape, the cells in the spheroids still seemed to hold together when we used PRIMA-1 at 50 μM, with cell viability reduced to 48.8% (Figure 7D). Spheroids fell apart in the treatment with 100 μM, including the necrotic zone of the spheroids, dropping viability to 24.9%. Thus, we can suggest that PRIMA-1 has an activity in preventing the formation and disrupting Huh-7 spheroids, which might be related to the adhesion inhibition reported previously in the colony formation assay (Figure 7B).

We then investigated whether PRIMA-1 is able to induce apoptosis in Huh-7 mutp53 cells. We used HepG2 cells as a control in order to compare PRIMA-1 effects on a WTp53 cell line. Cisplatin was used as a positive control of apoptosis induction. Our data show that PRIMA-1 did not promote much apoptotic or necrotic activity in HepG2-expressing WTp53, whereas cisplatin demonstrated approximately 43% initial apoptosis (Figure 7E). In Huh-7 cells, PRIMA-1 induced approximately 70% of initial apoptosis at the highest concentration tested (50 µM), while cisplatin achieved approximately 30%. Our combined data suggest that the PRIMA-1 reactivation of WTp53 in Huh-7 cells results in cell migration and colony formation inhibition. PRIMA-1 also prevents the formation and disrupts the 3D structure of spheroids while reducing their viability through apoptosis induction.

### PRIMA-1 and cisplatin synergize in p53-mutant HCC cells

**FIGURE 8:**
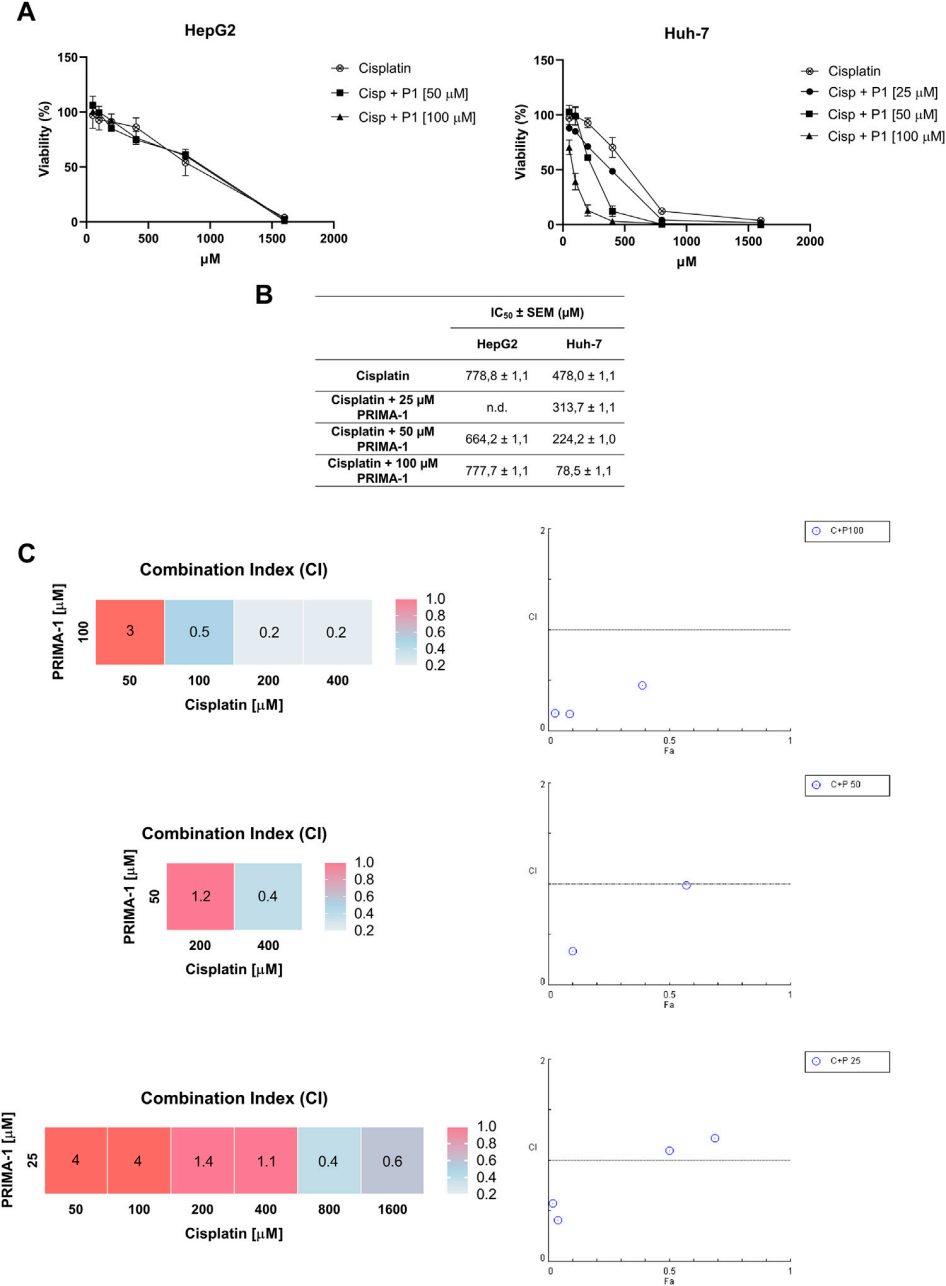
Combined PRIMA-1 (P1) and cisplatin (Cisp) chemosensitivity. **(A)** Cells were treated with different concentrations of cisplatin and PRIMA-1 (25, 50, and 100 µM) for 24 h and analyzed by MTT assay. **(B)** Calculation of the IC_50_ values was performed through dose-response curves. **(C)** Combination index (CI) was investigated using CompuSyn software, where a CI < 1 indicates synergism (blue), a CI = 1 indicates an additive effect (pink), and a CI > 1 indicates antagonism (red).

Cisplatin is a DNA-damaging agent that activates WTp53, thus inducing the transcription of critical p53 target genes in response to this damage (Basu and Krishnamurthy, 2010). We decided to explore whether treatment with PRIMA-1 would synergize with cisplatin in Huh-7 cells harboring the Y220C mutation of p53. Given that cisplatin is a commonly used drug in HCC chemotherapy, this may provide therapeutic benefits to a subset of HCC patients. Concomitant treatment with PRIMA-1, even at a concentration that did not affect cell viability alone (25 µM), promoted a reduction in cisplatin IC_50_ values in Huh-7 cells, while no effect was observed for HepG2 cells (Figures 8A, B). IC_50_ values corresponding to the treatment of HepG2 with cisplatin alone, cisplatin with 50 µM PRIMA-1, and cisplatin with 100 µM PRIMA-1 were 778.8 ± 1.1, 664.2 ± 1.1, and 777.7 ± 1.1 µM, respectively. Interestingly, the combination between the compounds showed enhanced activity in Huh-7 cells with IC_50_ values of 478.0 ± 1.1 µM (cisplatin), 313.7 ± 1.1 µM (cisplatin with 25 µM of PRIMA- 1), 224.2 ± 1.0 µM (cisplatin with 50 µM of PRIMA- 1), and 78.5 ± 1.1 µM (cisplatin with 100 µM PRIMA-1) (Figure 8B).

Given our findings that PRIMA-1 sensitizes Huh-7 cells to cisplatin treatment, we analyzed the effect of the combination of PRIMA-1 with cisplatin using CompuSyn software. The combinations of 25 µM PRIMA-1 with cisplatin showed antagonistic effects at concentrations of 50 and 100 μM, additive effects with 200 and 400 μM, and synergistic effects with 800 and 1,600 µM. With 50 µM of PRIMA-1, the effect was synergistic (400 µM) and additive (200 µM) with cisplatin. With 100 µM of PRIMA-1 and 50 µM of cisplatin, it was antagonistic, while 100, 200, and 400 µM showed synergism (Figure 8C). The synergistic effects seen are an indication of a possible clinical application of the combination of PRIMA-1 and cisplatin. Other reports have described the benefits of this combination *in vitro* and *in vivo*, with PRIMA-1 and PRIMA-1^MET^ in different tumor models (Bykov et al., 2005; Roh et al., 2011; Mssina et al., 2012; Kobayashi et al., 2013). Our findings, along with previously published data, provide a proof of concept for a novel combination therapy for the treatment of HCC.

Mutations in the *TP53* gene, along with a subsequent increase in the concentration of intracellular glutathione, can lead to platinum resistance. PRIMA-1 is a prodrug converted into methylene quinuclidinone (MQ), which binds to cysteine residues of mutp53, restoring the functional conformation of p53. In addition, this compound also binds to the cysteine residue in glutathione, thereby promoting a reduction in its concentration within the cell. Through this capacity, it results in the expansion of the apoptotic response and in the potentiation of the functioning of cisplatin (Mohell et al., 2015). For this reason, cotreatment with PRIMA-1 and cisplatin could trigger a reduction in chemotherapy doses, a reduction in side effects, and an improvement in patient survival (Izetti et al., 2014).

Our findings discussed herein provide, for the first time, evidence of amyloid-state p53 as an actionable therapeutic target in HCC. We characterize an HCC mutp53 cellular model for the study of p53 aggregation in cells from *in silico* analyses to a 3D-cell culture model and demonstrate the unprecedented inhibition of Y220C mutp53 aggregation by PRIMA-1. Furthermore, our data show the beneficial effects of PRIMA-1 in several cancer cell properties related to mutant p53 GoFs, including migration, adhesion, proliferation, and drug resistance. We also found the combination of PRIMA-1 and cisplatin as a promising approach for HCC therapy. Taken together, these results demonstrate that p53 amyloid aggregation is a potential pharmacological target for HCC, and PRIMA-1 may serve as a new candidate for combination therapy with cisplatin in HCC and possibly other tumor types.

## Data Availability

The raw data supporting the conclusions of this article will be made available by the authors, without undue reservation.
